# Supplementary material for: Rapidly receding Arctic Canada glaciers revealing landscapes continuously ice-covered for more than 40,000 years
Source: Nat Commun. 2019 Jan 25;10:445. doi: 10.1038/s41467-019-08307-w (PMC6347664; doi:10.1038/s41467-019-08307-w)
Supplement: Supplementary file 1 — Supplementary Information [file 41467_2019_8307_MOESM1_ESM.pdf]

Supplementary Information to Rapidly receding Arctic Canada glaciers revealing landscapes continuously ice-covered for more than 40,000 years

Pendleton* et al.

*Corresponding author email: simon.pendleton@colorado.edu

Supplementary Figures

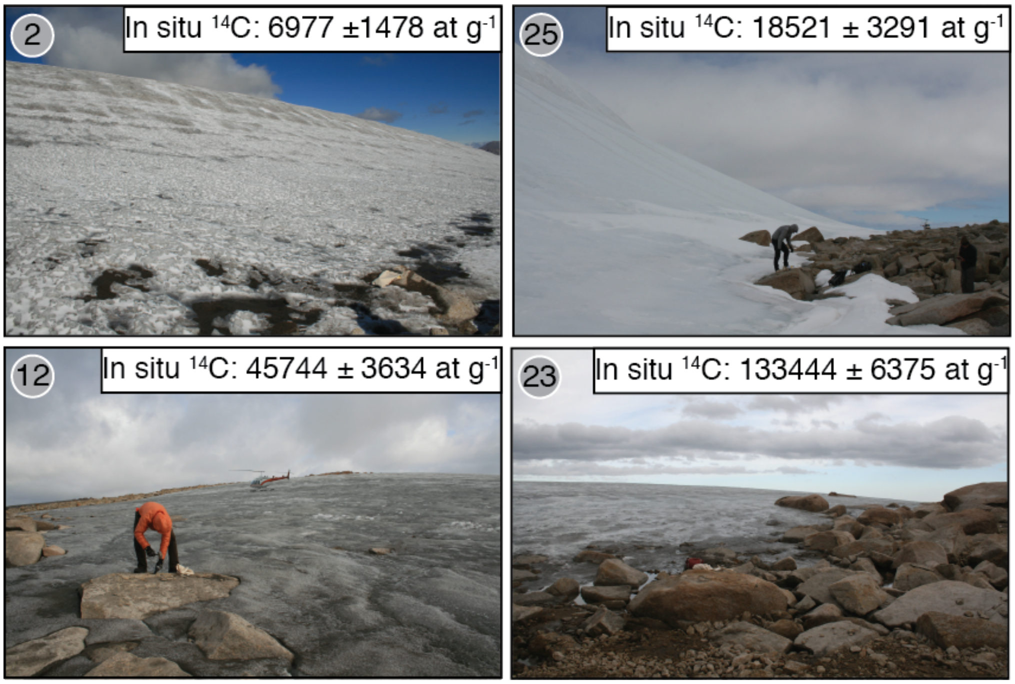

Supplementary Figure 1: Sample photos from four locations (circled numbers) where recently exposed rooted plants and preserved rock surfaces were sampled for ^{14}C analysis. These four locations represent the range of *in situ* ^{14}C inventories sampled in this study. The thick ice adjacent to site 25 suggests significant shielding during ice covered times, which is supported by the low inventory. Locations 12 and 23 both have relatively thin ice currently, but their varying inventories suggest different ice thickness histories during the Holocene. Location 2 has a significantly lower *in situ* ^{14}C inventory than the other samples, suggesting substantially more burial by thicker ice.

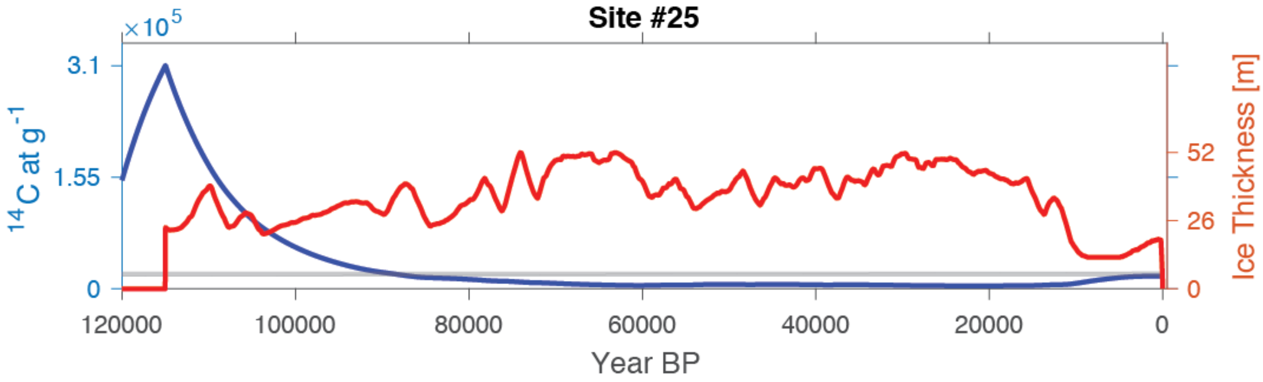

Supplementary Figure 2: Full simulation run from the last interglacial period (LIG) to present using conditions from location 25. Illustrates the decay of *in situ* ^{14}C accumulated during the LIG to background levels prior to the LGM. It also shows that the modern *in situ* ^{14}C inventory is primarily the result of thin ice cover during the Holocene (with a small amount accumulating during deglaciation).

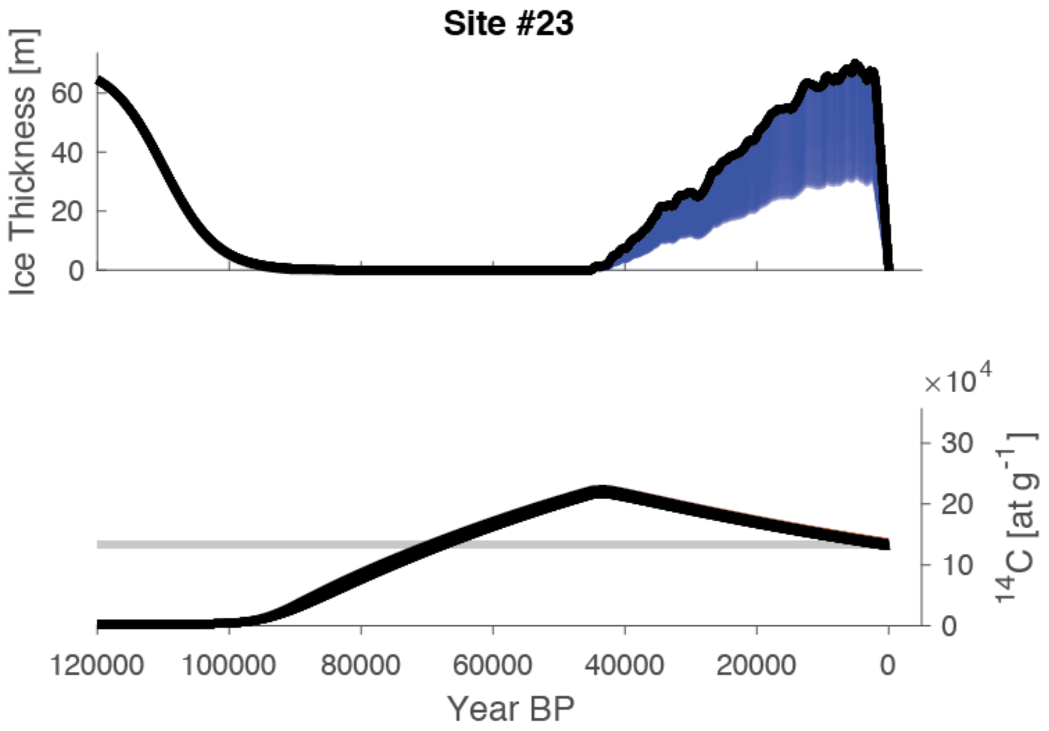

Supplementary Figure 3: Model output showing the last 12 kyr for site #23, the highest inventory site and the only one that can experience early-mid Holocene exposure and still decay back to an *in situ* ^{14}C concentration within uncertainty of the observed concentration. The top panel shows all possible Holocene exposure and subsequent Neoglacial burial scenarios (blue lines, solid black line is the median ice history), and the bottom panel are the resulting *in situ* ^{14}C concentrations (red lines, solid black line is the median *in situ* ^{14}C history; in this case all *in situ* ^{14}C histories are close together in concentration space).

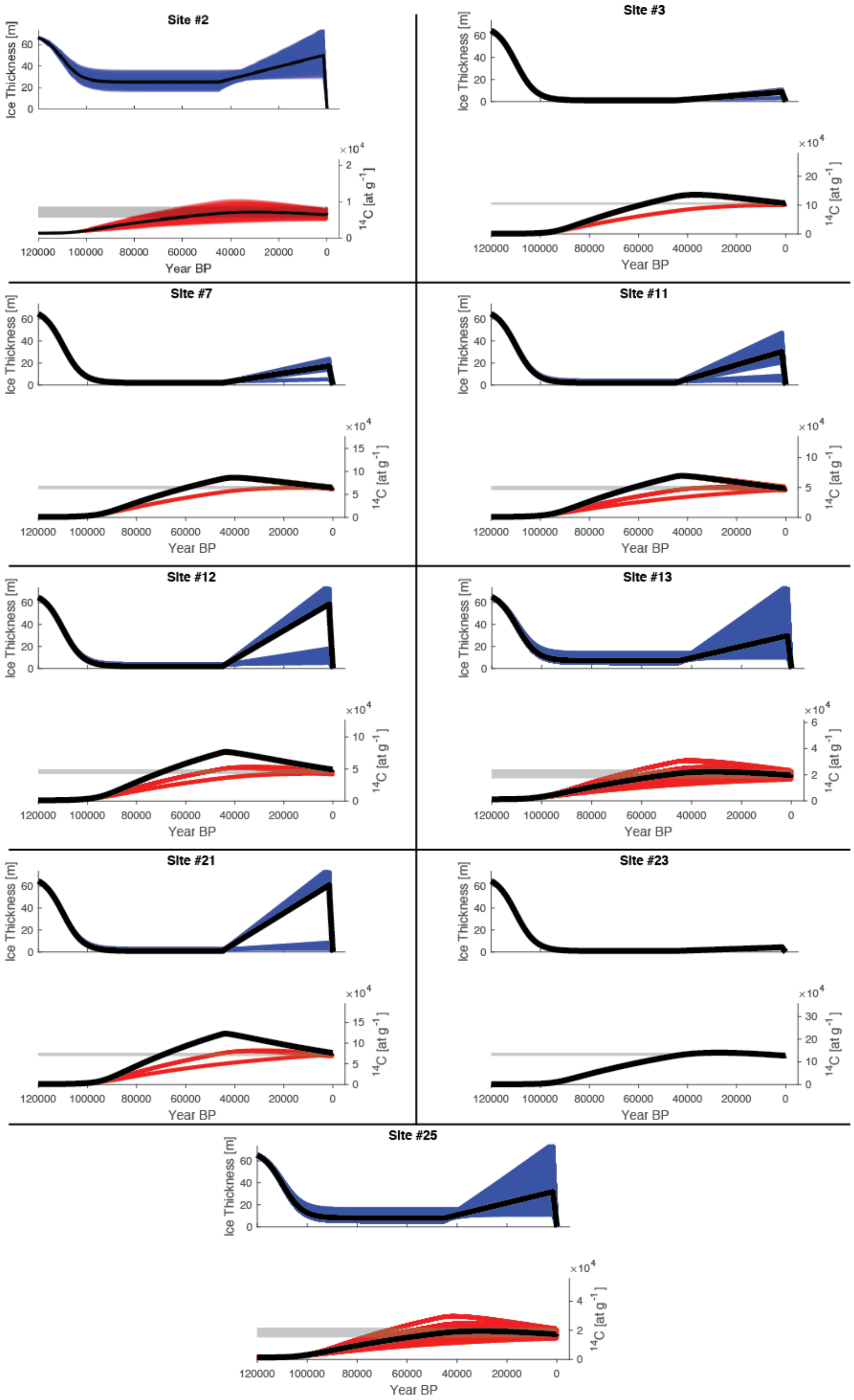

Supplementary Figure 4: Model output for all sample locations for the continuous Holocene ice cover scenarios. Each panel shows the possible Holocene ice cover histories (blue lines) and resulting *in situ* ^{14}C concentration histories (red lines); the median of both the ice histories and *in situ* ^{14}C inventories are shown by solid black lines.

Supplementary Tables

Supplementary Table 1

Samples with a > age had ^{14}C analyses indistinguishable from background. All other sample ages reported in calibrated years BP, using IntCal13 and Oxcal 4.2.4[1,2]

Site #	Lab ID	Sample ID	Material Dated	Latitude	Longitude	Altitude	^{14}C age (yr)	^{14}C ±1σ	Cal Age (yr)	±1σ
1	CURL-18239	M13-B002v	3 Moss Strands	67.6819	-65.8978	1298	>43300	-	-	-
2	CURL-17399	M13-B005v	6 Moss Strands	67.6049	-65.9705	1481	>48370	-	-	-
2	CURL-17812	M13-B007v	3 Moss Strands	67.6054	-65.9658	1469	>45277	-	-	-
3	CURL-17825	M13-B011v	2 Moss Strands	67.5388	-66.0999	1389	43770	4670	45443	+4557/-1177
3	CURL-18789	M14-B101v	2 Moss Strands	67.5438	-66.1178	1476	>46320	-	-	-
4	CURL-17808	M13-B018v	1 Moss Strand	67.4053	-66.1728	1397	>45277	-	-	-
5	CURL-17824	M13-B028v	3 Moss Strands	67.4533	-65.5646	1526	>45277	-	-	-
6	CURL-17391	M13-B045v	6 Moss Strands	67.6680	-65.6953	1687	>49990	-	-	-
7	CURL-17828	M13-B051v	3 Moss Strands	67.5622	-65.7534	1533	>45277	-	-	-
7	CURL-21559	M13-B052v	3 Moss Strands	67.5631	-65.7568	1550	>47000	-	-	-
7	CURL-18792	M14-B139v	3 Moss Strands	67.5607	-65.7525	1524	>44940	-	-	-
8	CURL-17837	M13-B055v	6 Moss Strands	67.5017	-65.5399	1589	>45277	-	-	-
9	CURL-18265	M13-B064v	1 Moss Strand	67.4988	-65.0401	1431	41800	3250	45171	+2893/-2420
10	CURL-17390	M13-B066v	6 Moss Strands	67.8415	-66.3015	1519	45830	1770	48199	+1801/-520
10	CURL-18249	M13-B069v	2 Moss Strands	67.8398	-66.2994	1505	>47800	-	-	-
11	CURL-17403	M13-B094v	1 Moss Strand	67.9618	-66.6450	1256	48850	2570	48491	+1509/-390
11	CURL-23040	M13-B091v	1 Moss Strand	67.9621	-66.6432	1246	45240	2570	47449	+2551/-730
11*†	UCIAMS-84687	M10-B258v	Fruticose Lichen	67.9618	-66.6452	1261	34300	3600	38214	+3662/-3200
11*†	UCIAMS-85577	M10-B258v	Fruticose Lichen	67.9618	-66.6452	1261	39740	950	43550	+689/-810
11*†	UCIAMS-85969	M10-B258v	Fruticose Lichen	67.9618	-66.6452	1261	37510	490	41880	+380/-320
12	CURL-17815	M13-B104v	3 Moss Strands	67.5500	-64.9203	1390	>45277	-	-	-
12*†	UCIAMS-84673	M10-B231v	Rooted Moss Strands	67.5491	-64.9208	1395	29100	1500	33094	+1265/-1600
12*†	UCIAMS-85576	M10-B231v	Rooted Moss Strands	67.5491	-64.9208	1395	44300	1300	47570	+1306/-1280
12*†	UCIAMS-85968	M10-B231v	Rooted Moss Strands	67.5491	-64.9208	1395	23920	100	27959	+97/-150
12*†	UCIAMS-84674	M10-B232v	Rooted Moss Strands	67.5492	-64.9218	1396	37500	3600	41194	+3738/-3110
13	CURL-17393	M13-B195v	3 Moss Strands	67.0174	-64.3486	1589	52120	3860	48226	+1774/-460
13	CURL-21586	M13-B196v	3 Moss Strands	67.0176	-64.3492	1587	42100	1270	45545	+1080/-1280
14	CURL-17426	M13-B201v	6 Moss Strands	66.9875	-64.5361	1506	50300	3080	48419	+1581/-410
15	CURL-18761	M14-B020v	3 Moss Strands	67.4331	-65.3250	1584	>45650	-	-	-
16	CURL-18801	M14-B085v	3 Moss Strands	67.6212	-66.1851	1417	39280	1230	43228	+910/-980
17	CURL-18784	M14-B107v	6 Moss Strands	67.5662	-65.2447	1293	>46320	-	-	-
18	CURL-18805	M14-B113v	4 Moss Strands	67.4933	-65.0134	1520	>46320	-	-	-
19	CURL-18785	M14-B143v	2 Moss Strands	67.5455	-65.6447	1502	>46320	-	-	-
20	CURL-18819	M14-B154v	5 Moss Strands	66.8027	-64.5740	1585	>45980	-	-	-
21	CURL-18791	M14-B158v	1 Moss Strand	67.5345	-64.8350	1386	>46320	-	-	-
22	CURL-18796	M14-B163v	3 Moss Strands	67.4905	-65.6690	1582	>46380	-	-	-
23	CURL-18803	M14-B164v	1 Moss Strand	67.5644	-64.9401	1441	>45220	-	-	-
23	CURL-23056	M14-B165V	1 Moss Strand	67.5643	-64.9382	1432	46120	2870	47592	+2408/-690
24	CURL-23060	M14-B183V	3 Moss Strands	67.0324	-65.1152	1573	45780	2750	47549	+2451/-700
24	CURL-18812	M14-B184v	3 Moss Strands	67.0323	-65.1157	1576	>46320	-	-	-
25	CURL-21555	M15-B047v	3 Moss Strands	66.9871	-65.3017	1526	>47000	-	-	-
25	CURL-20150	M15-B048v	2 Moss Strands	66.9873	-65.3002	1531	>44400	-	-	-
26*†	UCIAMS-84681	M10-B247v	Fruticose Lichen	67.7047	-64.7109	929	45600	2500	47636	+2364/-680
27*†	UCIAMS-84686	M10-B255v	Fruticose Lichen	68.0062	-66.6180	1092	43200	2700	46338	+2541/-1830
27*†	UCIAMS-123039	M10-B256v	Rooted Moss Strands	68.0062	-66.6180	1092	50700	3100	48468	+1532/-390
28	CURL-23359	M14-B009v	3 Moss Strands	67.3247	-65.3766	1543	44200	1850	47303	+1842/-1370
29	CURL-23332	M13-B046v	3 Moss Strands	67.6165	-65.7000	1300	>50143	-	-	-
30	CURL-23347	M14-B161v	3 Moss Strands	67.5238	-64.9978	1352	>50768	-	-	-

* from Miller et al. (2013)

†Received only DI pretreatment

Supplementary Table 2

The sample ID, latitude, longitude, elevation, and *in situ* ^{14}C concentrations and associated process blanks at 9 of the 30 sites sampled for *in situ* ^{14}C and the results ice cover simulations. Only the high inventory at site 23 allows for Holocene exposure (maximum allowable duration shown). Under the imposed ice cover scenario, no other site can be ice free during the early-mid Holocene. *In situ* ^{14}C can be reproduced at all sites with continuous ice cover, although thin, through the middle Holocene. Sample a is a steep-sided summit that has likely never been glaciated, and so is not included in the ice cover simulations but included for inventory comparison.

										Continuous Holocene Coverage	
**Site #**	**Sample ID**	**Latitude**	**Longitude**	**Elevation (m)**	**^{14}C Conc. (at g^{-1})****	**± (at g^{-1})**	**Lab Blank Conc. (at)**	**± (at)**	**Max. Exposure prior to 4.5 ka (kyr)**	**Holocene Ice Thickness (m)**	**Peak LIA Ice Thickness (m)**
2	M14-B090R	67.60514	-65.96841	1478	6977	1491	160700	12100	0	26 (18 - 36)	50 (29 - 70)
3	M14-B098R	67.54543	-66.12091	1478	105786	3969	303500	30900	0	1 (1 - 2)	9 (3 - 11)
7	M14-B140R	67.56106	-65.75166	1522	65206	3530	401000	30900	0	2 (2 - 3)	18 (5 - 24)
11	M13-B095R	67.96188	-66.64453	1255	49267	3523	297600	30900	0	2 (2 - 3)	33 (6 - 49)
12	M13-B107R	67.55004	-64.92057	1389	45744	3634	401000	30900	0	3 (2 - 4)	17 (5 - 70)
13	M13-B198R	67.01746	-64.34822	1588	20867	3384	401000	30900	0	6 (4 - 13)	29 (10 - 70)
21	M14-B159R	67.53451	-64.83459	1390	72950	3696	154200	30900	0	1 (1 - 2)	66.5 (6 - 70)
23	M14-B166R	67.5644	-64.94101	1436	133444	6375	276900	30900	5	1 (1 - 1)	4 (4 - 4)
25	M15-B046R	66.98708	-65.30172	1526	18521	3291	154200	30900	0	7 (4 - 15)	29 (11 - 70)
a*	M14-B001R	67.66932	-64.26317	1010	368762	7090	380600	30900	N/A	N/A	N/A

*Coastal summit bedrock under constant exposure (Holocene ice cover simulations inappropriate), reported for comparison.

**10g of sample was dissolved for each analysis

Supplementary Table 3

In situ ^{14}C production rates from this and other studies on Baffin Island

Source	Sample ID	Latitude	Longitude	Elevation (m asl)	Sample Thickness (cm)	Sample Density (g cm^{-3})	Shielding Correction	Erosion (cm yr^{-1})	[^{14}C] (at g^{-1})	± (at g^{-1})	Production rate[a] (at g^{-1} yr^{-1})
Anderson et al., 2008	05ORN-04	71.5875	-78.1960	819	5	2.7	1	0	85900	4200	34
	05ORN-05	71.5875	-78.1960	819	3	2.7	1	0	88900	4100	34
	05ORN-32	71.5425	-78.0361	888	3	2.7	1	0	114700	4400	36
	05SRP-15	71.4753	-77.5129	797	4	2.7	1	0	106600	4200	34
	05TGR-08	71.3650	-78.7802	741	1	2.7	1	0	126900	5100	32
	06ORN-20	71.6370	-78.0894	760	1	2.7	1	0	138400	4900	33
Miller et al., 2006	CR04-12	69.6290	-70.8800	939	1	2.7	1	0	248900	8880	38
This study	M14-B090R	67.6051	-65.9684	1478	1	2.7	1	0	7000	1500	59
	M14-B098R	67.5454	-66.1209	1478	1	2.7	1	0	105800	4000	58
	M14-B140R	67.5611	-65.7517	1522	1	2.7	1	0	65200	3500	62
	M13-B095R	67.9619	-66.6445	1255	1	2.7	1	0	49300	3500	49
	M13-B107R	67.5500	-64.9206	1389	1	2.7	1	0	45700	3600	55
	M13-B198R	67.0175	-64.3482	1588	1	2.7	1	0	20900	3400	65
	M14-B159R	67.5345	-64.8346	1390	1	2.7	1	0	73000	3700	55
	M14-B166R	67.5644	-64.9410	1436	1	2.7	1	0	133400	6400	57
	M15-B046R	66.9871	-65.3017	1526	1	2.7	1	0	18500	3300	61
	M14-B001R	67.6693	-64.2632	1010	1	2.7	1	0	368800	7100	37

[a]*In situ* ^{14}C production rates are calculated using the version 3.0 MATLAB code of the CRONUS-Earth online calculator[3] (http://hess.ess.washington.edu) using a time-integrated production rate and the LSDn scaling framework[4–7].

Supplementary Discussion

Samples from the majority of the 30 locations have ^{14}C concentrations indistinguishable from background at two-sigma and are given as age limits. In addition, three samples have technically finite ages larger than 50,000 ^{14}C yrs. However, had we specified a background uncertainty slightly larger than is conventional (i.e., 30% at 1 sigma), these samples would have been indistinguishable from background at 2-sigma. It is therefore likely that these dates also represent minimum age limits. Several of the collections from the previous study returned pre-Holocene ages[8], however, those samples were only rinsed in deionized (DI) water, and trace levels of wind-blown modern organic debris may not have been removed during pretreatment. We subsequently pretreated those subsamples of same collections with repeated sonication in DI water and acid-base-acid (ABA) treatment, after which they returned ages closer to the limits of ^{14}C dating (Supplementary Table 1). All radiocarbon dates obtained from *in situ* plants following ABA pretreatment from these 30 unique ice caps returned ages >40 cal ka.

Supplementary References

1. Bronk-Ramsey, C. Bayesian Analysis of Radiocarbon Dates. *Radiocarbon* **51,** 337–360 (2009).
2. Reimer, P. J. *et al.* IntCal13 and Marine13 Radiocarbon Age Calibration Curves 0–50,000 Years cal BP. *Radiocarbon* **55,** 1869–1887 (2013).
3. Balco, G., Stone, J. O., Lifton, N. A. & Dunai, T. J. A complete and easily accessible means of calculating surface exposure ages or erosion rates from 10Be and 26Al measurements. *Quat. Geochronol.* **3,** 174–195 (2008).
4. Borchers, B. *et al.* Geological calibration of spallation production rates in the CRONUS-Earth project. *Quat. Geochronol.* **31,** 188–198 (2016).
5. Phillips, F. M. *et al.* The CRONUS-Earth Project: A synthesis. *Quat. Geochronol.* **31,** 119–154 (2016).
6. Lifton, N. Implications of two Holocene time-dependent geomagnetic models for cosmogenic nuclide production rate scaling. *Earth Planet. Sci. Lett.* **433,** 257–268 (2016).
7. Balco, G. Production rate calculations for cosmic-ray-muon-produced ^{10}Be and ^{26}Al benchmarked against geological calibration data. *Quat. Geochronol.* **39,** 150–173 (2017).
8. Miller, G. H., Lehman, S. J., Refsnider, K. A., Southon, J. R. & Zhong, Y. Unprecedented recent summer warmth in Arctic Canada. *Geophys. Res. Lett.* **40,** 5745–5751 (2013).
